# Involvement of Toll-like receptor 2 in the cerebral immune response and behavioral changes caused by latent *Toxoplasma* infection in mice

**DOI:** 10.1371/journal.pone.0220560

**Published:** 2019-08-12

**Authors:** Fumiaki Ihara, Sachi Tanaka, Ragab M. Fereig, Maki Nishimura, Yoshifumi Nishikawa

**Affiliations:** 1 National Research Center for Protozoan Diseases, Obihiro University of Agriculture and Veterinary Medicine, Inada-cho, Obihiro, Hokkaido, Japan; 2 Department of Animal Medicine, Faculty of Veterinary Medicine, South Valley University, Qena City, Qena, Egypt; Karolinska Institutet, SWEDEN

## Abstract

Subacute and chronic infections with the intracellular protozoan parasite *Toxoplasma gondii* are associated with an increased risk of psychiatric diseases like schizophrenia. However, little is known about the mechanisms involved in *T*. *gondii*-induced neuronal disorders. Recently, we reported that Toll-like receptor 2 (TLR2) was required to initiate the innate immune response in cultured mouse brain cells. However, how TLR2 contributes to latent infection with *T*. *gondii* remains unclear. Therefore, we examined the role of TLR2 in brain pathology and behavior using wild-type (TLR2^+/+^) and TLR2-deficient (TLR2^-/-^) mice. The behavioral analyses showed that TLR2 deficiency increased the anxiety state of the uninfected and infected animals alike, and TLR2 deficiency showed no relationship with the infection. In the contextual and cued fear-conditioning tests, *T*. *gondii* infection decreased the mouse freezing reaction while TLR2 deficiency increased it, but there was no interaction between the two factors. Our histopathological analysis showed that the TLR2^+/+^ and TLR2^-/-^ mice had similar brain lesions at 30 days post infection (dpi) with *T*. *gondii*. Higher numbers of parasites were detected in the brains of the TLR2^-/-^ mice than in those from the TLR2^+/+^ mice at 30 dpi, but not at 7 and 14 dpi. No significant differences were observed in the proinflammatory gene expression levels in the TLR2^+/+^ and TLR2^-/-^ mice. Therefore, it appears that TLR2 signaling in the brain might contribute to the control of parasite growth, but not to brain pathology or the impaired fear memory response induced by infection with *T*. *gondii*.

## Introduction

Until now, most studies on the roles played by Toll-like receptors (TLRs) have been focused on the immune system, but it is now known that TLRs also play important roles in neurodevelopment, adult neurogenesis, neuroplasticity, as well as in learning and memory [1]. The possible roles for TLR2 in cognition await determination but seem predictable given the evidence that TLR2 deficiency impairs hippocampal neurogenesis in mice [2]. A recent study showed that TLR2 deficiency in mice induces some of the behaviors associated with schizophrenia, such as hyperactivity, social withdrawal, and cognitive impairment [3]. Moreover, the same study showed that TLR2^-/-^ mice had ventricle enlargement and dysregulation of the Akt-GSK-3α/β signaling pathway, which is associated with the pathogenesis of numerous neurological and psychiatric disorders [3].

The effect of TLR2 on mouse survival during an infection with *T*. *gondii* is both dose and parasite strain dependent [4–7]. Mun et al. (2003) reported that 0%, 80%, and 100% of TLR2^-/-^ mice survived after infection with 300 cysts, 100 cysts, and 50 cysts from the *T*. *gondii* Fukaya strain, respectively, while all C57BL/6 mice survived this infection. Debierre-Grockiego et al. (2007) also showed that all TLR2^-/-^ mice and 80% of TLR2^+/+^ mice survived their infections with 10 cysts from the ME49 strain. Thus, considering the results from the above-mentioned studies, TLR2 is probably not involved in mouse survival against infection with a non-lethal dose of *T*. *gondii*. However, in contrast with mouse survival, TLR2 plays an important role in inhibiting parasite activity in the brain.

A previous study showed that brain parasite numbers in TLR2^-/-^ mice were higher than those of the TLR2^+/+^ mice at 8 days post-intraperitoneal infection with 300 cysts from the Fukaya strain [6]. However, there were no differences between the TLR2^+/+^ and TLR2^-/-^ mice at 40 days post intraperitoneal infection with 10 cysts of the ME49 strain [5]. The increased parasite burdens at the acute stage (0–2 weeks after the infection) and subacute stage (3 to 5 weeks after the infection) might result from impaired peripheral immunity levels. Generally, IL-12 produced from innate immune cells such as macrophages and dendritic cells at the peripheral infection sites induces IFN-γ production by T-helper 1 cells and promotes the development of cytolytic CD8^+^ T cells, resulting in control of *T*. *gondii* dissemination in the body [8]. Our previous study showed that IL-6 and IL-12p40 production from *T*. *gondii*-infected TLR2^-/-^ mouse peritoneal macrophages decreased when compared with the infected TLR2^+/+^ cells [9]. Additionally, lower levels of IFN-γ and IL-12 were detected in the peritoneal exudate cells from TLR2^-/-^ mice than in the same cells from TLR2^+/+^ mice at day 5 post infection [6], resulting in an increased parasite burden in the lungs of TLR2^-/-^ mice intraperitoneally injected with 100 cysts from the Fukaya strain [4]. Thus, compared with the TLR2^+/+^ mice, the tachyzoites that escaped periphery immunity in the TLR2^-/-^ mice may have reached the CNS during the acute infection stage.

We recently showed that TLR2 regulates many genes in primary cultured astrocytes, microglia, and neurons during *Toxoplasma gondii* infection, especially the innate immune response genes, suggesting that TLR2 plays an important role in host resistance to CNS infections with *T*. *gondii* [9]. However, the role played by TLR2 signaling in the chronic stage of infection with *T*. *gondii* (more than 6 weeks after the infection) awaits discovery. It is believed that latent toxoplasmosis is asymptomatic in healthy adults, but recently epidemiological studies have revealed a link between *Toxoplasma* infection and the development of mental illnesses such as schizophrenia [10–13]. Furthermore, latent toxoplasmosis is associated with personality changes and suicide in humans [14,15], and an increasing number of studies have found that this infection is correlated with an array of host behavioral changes [16]. A number of studies have found that rodents infected with *T*. *gondii* exhibit decreased avoidance behavior in response to cat odors, indicating that this parasite can also manipulate host behavior [17–21]. Moreover, we previously reported that infection with *T*. *gondii* impaired fear memory consolidation in mice [22].

Consequently, we hypothesized that TLR2 plays a critical role in *T*. *gondii*-induced neuronal disorders. Therefore, we investigated the effects of TLR2 deficiency on the neuro-immunological and behavioral changes that occur during chronic infections with *T*. *gondii*. To assess the above hypothesis, mice were inoculated with a non-lethal dose of parasites in the present study because high-doses cause clinical manifestations such as severe hypoactivity, making it difficult to accurately compare the results of the behavioral tests.

## Material and methods

### Ethics statement

The use and care of animals complied with the Guide for the Care and Use of Laboratory Animals from the Ministry of Education, Culture, Sports, Science, and Technology, Japan. The protocol was approved by the Committee on the Ethics of Animal Experiments at the Obihiro University of Agriculture and Veterinary Medicine (permit number: 29–61 and 29–62). All efforts were made to minimize animal suffering.

### Animals

TLR2-deficient (TLR2^-/-^) mice were kindly supplied by Drs. Satoshi Uematsu and Shizuo Akira (Osaka University, Japan) [23]. C57BL/6J mice, 6–8 weeks of age from Clea Japan (Tokyo, Japan) were used as controls for the TLR2^-/-^ mice because they have the same genetic background, except for the TLR2 mutation. The animals were housed under specific-pathogen-free conditions in the animal facility of the National Research Center for Protozoan Diseases at Obihiro University of Agriculture and Veterinary Medicine, Obihiro, Japan (NRCPD). As the breeding environment might affect the behavior of the mice, they were bred in the animal faculty of the NRCPD, and the offspring were used in the behavioral experiments. Mice were sacrificed by cervical dislocation or decapitation under deep anesthetization with isoflurane. Altogether, 115 mice were used throughout this study.

### Parasite culture

The type II PLK strain, which was kindly gifted by Dr. Kami Kim (Albert Einstein College of Medicine), was passaged in monkey kidney adherent epithelial cells (Vero cells) (ATCC: CCL-81) in Eagle’s minimum essential medium (Sigma, St. Louis, MO, USA) with 8% fetal bovine serum and antibiotics. Infected cells were syringe-lysed using a 27-gauge needle to release the tachyzoite-stage parasites into RPMI-1640 medium (Sigma), which was then filtered using a 5.0-μm pore-sized filter (Millipore, Bedford, MA, USA).

### Parasite infections

*T*. *gondii* tachyzoites were intraperitoneally inoculated (1 × 10^3^ tachyzoites) into 9-week-old male mice after allowing 1 week of environmental adjustment. Daily body weight measurements were taken for 4 weeks after infection. All the behavioral experiments were performed at 30–41 days post infection (dpi), at 7:00–8:30 a.m. under a light intensity of 300 lux.

### Open-field test

To investigate activity and exploratory behavior in *T*. *gondii*-infected mice, the mice were tested one at a time. As described in detail previously [24], exploration in an open field, a circular area with a diameter of 50 cm (Muromachi, Tokyo, Japan), was recorded for 5 min using a video tracking system (Comp Act VAS ver. 3.0x, Muromachi). Total travelled distance (cm/5 min), average speed (cm/sec), and first response latency (sec) were measured. The behavioral experiments were performed on day 30 post infection, and commenced at 7:00 am, under 300 lux light intensity.

### Hole-board test

Exploration in a hole-board, a square area equipped with four holes with a side length of 50 cm (Muromachi), was recorded for 5 min with a video tracking system (Comp Act VAS ver. 3.0x). The latency time to first head-dipping (sec), head-dipping count (counts/5 min), and head-dipping duration (sec/5 min) was measured. Behavioural experiments were performed on day 31 post infection, and commenced at 7:00 am, under 300 lux light intensity.

### Fear-conditioning test

To assess the ability of the mice to develop a fear memory, a contextual and cued fear-conditioning experiment was performed in a fear-conditioning box (18 cm × 17 cm; Muromachi). The experimental conditions have been described in detail previously [22]. Briefly, the fear-conditioning test was conducted over 4 days. On day 1, once placed in a conditioning chamber, each mouse received a paired auditory cue and mild foot shock twice. On day 2, to test the contextually-conditioned fear memory, the mice were again individually placed in the same conditioning chamber and tested for 5 min without the foot shocks and without tone cue (context test). On day 3, to test for a conditioned fear of the tone cue in the absence of contextual cues, the individual mice were placed in a differently shaped chamber for habituation over 3 min, and the auditory cue was presented for 3 min (tone test). Freezing behavior, measured as an index of fear memory, was recorded using a video-tracking system (Comp Act VAS ver. 3.0x). On day 4, to test the ability of the mice to extinct the conditioned fear memory, the individually-tested mice received 30 successive auditory cues without the foot shock (extinction test). Freezing was measured over 5 min in the context test, during habituation (3 min), and during the tone (3 min) in the tone test. The freezing ratio (%) was calculated by dividing the time spent freezing by the total time of each session. In the extinction test, the freezing ratio (%) was repeatedly calculated by dividing the time spent freezing by each 5-min extinction test. It must be noted that the wild-type mice were the same as those used in our previous study because the behavioral experiment in this study was conducted at the same time as the experiments described in a previous report [22]. In our previous study, behavioral experiments consisted of 4 independent experiments. Data belonging to TLR2^-/-^ mice were collected in parallel to the latter half of the 4 experiments. Although behavioral studies were performed under non-blind test, it had no opportunity for any arbitrary count because all data were collected in automatically by the video-tracking system.

### Pathological analysis

The pathological analysis of the mice was performed as described in detail previously [25]. After fixation with 10% neutral-buffered formalin solution, the coronally-cut mouse brains were routinely embedded in paraffin wax, sectioned at 4 μm, and stained with hematoxylin and eosin. To estimate the severity of the histopathological lesions, they were scored as follows: 0, no lesions; 1, mild lesions limited to localized perivascular cuffs, or slight mononuclear cell infiltration of the meninges and slight glial cell infiltration; 2, moderate lesions, including multiple perivascular cuffs, meningitis, and local glial cell infiltration; 3, severe lesions, including multiple or widespread perivascular cuff, meningitis, glial cell activation, focal necrosis, and rarefaction of the neuropil, with occasional macrophage infiltration. The severity of the brain lesions was estimated in three sections cut at the caudate putamen, hypothalamus and cerebellum level. The pathological lesions representing the different scores are shown in S1 Fig The scores for each section were calculated, and the total pathological score for each mouse was used in the data analysis. We also counted the number of cysts in each section, as indicated in S1 Fig, and the total number of cysts for each mouse was used in the data analysis.

### DNA isolation and PCR analysis

DNA from the brain samples collected at 30 dpi was prepared. The DNA was extracted, purified, and quantified for parasite counts by real-time PCR using the B1 gene as previously described in detail [26]. PCR was performed using the ABI Prism 7900HT sequence detection system (Applied Biosystems, Foster City, CA, USA), and amplification was monitored using the SYBR green method (Applied Biosystems). The calculated cycle threshold (Ct) values were exported to Microsoft Excel for analysis. A standard curve was constructed with the *T*. *gondii* DNA extracted from 1 × 10^5^ parasites using 1 μl of a serial dilution ranging from 10,000 to 0.01 parasites. Parasite numbers were calculated by interpolation of a standard curve on which the Ct values were plotted against known concentrations of parasites. To confirm the specificity of the PCR, DNAs from the brain of an uninfected mouse and from purified *T*. *gondii* tachyzoites were used as the negative and positive controls, respectively.

### Quantitative reverse-transcription-PCR

mRNA expression levels in the mouse brains were measured at 30 dpi as previously described in detail [27]. Total RNA was extracted from the mouse brain samples using TRI Reagent (Sigma), and reverse transcription was performed using Superscript III^TM^ Reverse Transcriptase (Invitrogen, Life Technologies, Carlsbad, CA, USA), according to the manufacturer’s instructions. Expression of the genes encoding inducible nitric oxide synthase (iNOS), interleukin-12p40 (IL-12p40), IFN-γ, interleukin-6 (IL-6), interleukin-10 (IL-10), tumor necrosis factor-α (TNF-α), and glyceraldehyde 3-phosphate dehydrogenase (GAPDH) as a housekeeping gene was analyzed by real-time PCR using the Applied Biosystems Prism 7700 Sequence Detection System with SYBR Green master mix (Applied Biosystems). In the preliminary study, we compared the stability of the expression levels of several housekeeping genes such as GAPDH, beta-actin, and 18S ribosome RNA, and confirmed that GAPDH was the most appropriate housekeeping gene in brain tissue. The comprehensive gene stability tests were calculated by Reffinder (https://www.heartcure.com.au/for-researchers/). The relative gene stabilities were GAPDH = 1.00, 18s ribosome RNA = 1.682, and β-actin = 3.00. The sequences were as follows: iNOS: 5′-ACC CCT GTG TTC CAC CAG GAG ATG TTG AA-3′ (sense) and 5′-TGA AGC CAT GAC CTT TCG CAT TAG CAT GG-3′ (anti-sense); IL-12p40: 5′-GGA TGG AAG AGT CCC CCA AA-3′ (sense) and 5′- CTG GAA AAA GCC AAC CAA GC-3′ (anti-sense); IFN-γ: 5′- AGC TCA TCC GAG TGG TCC AC-3′ (sense) and 5′- GCT TCC TGA GGC TGG ATT CC-3′ (anti-sense); IL-6: 5′- TTC CAT CCA GTT GCC TTC TTG-3′ (sense) and 5′- GAA GGC CGT GGT TGT CAC C-3′ (anti-sense); IL-10: 5′- CCT GGT AGA AGT GAT GCC CC-3′ (sense) and 5′- TCC TTG ATT TCT GGG CCA TG-3′ (anti-sense); TNF-α: 5′- GGC AGG TCT ACT TTG GAG TCA TTG C-3′ (sense) and 5′- ACA TTC GAG GCT CCA GTG AA-3′ (anti-sense); and GAPDH: 5′- GGA GGC CAC ACT GCT GAT TTA-3′ (sense) and 5′- CCT GCT TCA CCA CCT TCT TGA T-3′ (anti-sense). The fold change C_t_ method was used, where C_t_ is the threshold concentration (User Bulletin no. 2; Perkin- Elmer, Boston, MA, USA).

### Statistical analyses

Statistical analyses were performed using GraphPad Prism (version 6.0) software (GraphPad Software, San Diego, CA, USA). Statistically significant differences between the different mouse groups (TLR2^+/+^ infected, TLR2^+/+^ uninfected, TLR2^-/-^ infected, TLR2^-/-^ uninfected) in the different experiments were analyzed using unpaired *t* tests and the Mann-Whitney test. Statistically significant differences among four groups were determined using two-way ANOVA with *Toxoplasma* infection and TLR2 deficiency as the main factors. *P* values of < 0.05 represent statistically significant differences.

## Results

### Open-field testing on *T*. *gondii*-infected and uninfected mice

**Fig 1:**
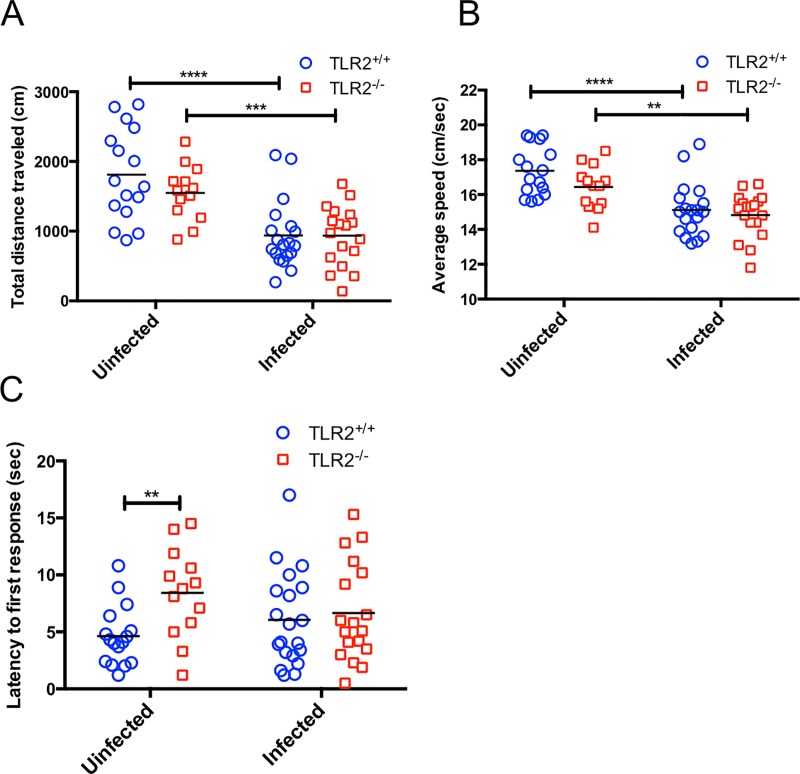
Effects of *T*. *gondii* infection on the locomotor activity of mice. Changes in mouse locomotor activity on day 30 post infection: (A) total distance travelled (cm), (B) average speed (cm/sec), and (C) latency to first response (sec). Data were summarized from two independent experiments (uninfected TLR2^+/+^ mice, n = 16; uninfected TLR2^-/-^ mice, n = 13; infected TLR2^+/+^ mice, n = 21; infected TLR2^-/-^ mice, n = 18). Significant differences among the four groups were analyzed by a two-way ANOVA followed by t tests (**, *p* < 0.01; ***, *p* < 0.001; ****, *p* < 0.0001), significant main effects are shown for (A) *T*. *gondii* infection [*F*_(1,62)_ = 35.42, *p* < 0.0001], (B) *T*. *gondii* infection [*F*_(1,62)_ = 31.85, *p* < 0.0001], and (C) TLR2 deficiency [*F*_(1,63)_ = 7.596, *p* < 0.05]. The interaction between *T*. *gondii* infection and TLR2 deficiency was not statistically significant.

To assess whether infection with *T*. *gondii* induced behavioral alternations in the TLR2^-/-^ mice, we conducted the following behavioral experiments: the open-field test, the hole-board test, and the fear conditioning test. First, the TLR2^+/+^ and TLR2^-/-^ mice were infected with the *T*. *gondii* PLK strain, and their bodyweights were measured daily for 4 weeks after infection. When compared with the uninfected mice, both the infected TLR2^+/+^ and infected TLR2^-/-^ mouse groups experienced significantly reduced body weights from 9 days after infection with *T*. *gondii*, but no mice died (S2 Fig). In the open-field test, the total distance travelled and average speed showed no difference between the TLR2^+/+^ and the TLR2^-/-^ uninfected mice (Fig 1A and 1B). However, the uninfected TLR2^-/-^ mice showed a significant increase in the latency to the first response compared with the uninfected TLR2^+/+^ mice (*p* < 0.01) (Fig 1C). This result suggests that TLR2-deficiency caused increased anxiety in the TLR2^-/-^ mice because they experienced slower initial movement times than those of the TLR2^+/+^ mice. Next, we assessed whether the absence of the TLR2 gene affected the behavioral changes caused by subacute infection with *T*. *gondii*. The total distance travelled and average speed of the infected mice decreased significantly when compared with the uninfected mice for both TLR2^+/+^ and TLR2^-/-^ mouse groups (Fig 1A and 1B). The two-way ANOVA analysis revealed significant effects for the factor ‘*Toxoplasma* infection’, indicating that mice infected with *T*. *gondii* experienced significantly decreased locomotion regardless of their TLR2^+/+^ or TLR2^-/-^ status. In addition, unlike the case of the uninfected animals, there was no significant difference in the latency to the first response between the infected TLR2^+/+^ and infected TLR2^-/-^ mice (Fig 1C).

### Hole-board testing on *T*. *gondii*-infected and uninfected mice

**Fig 2:**
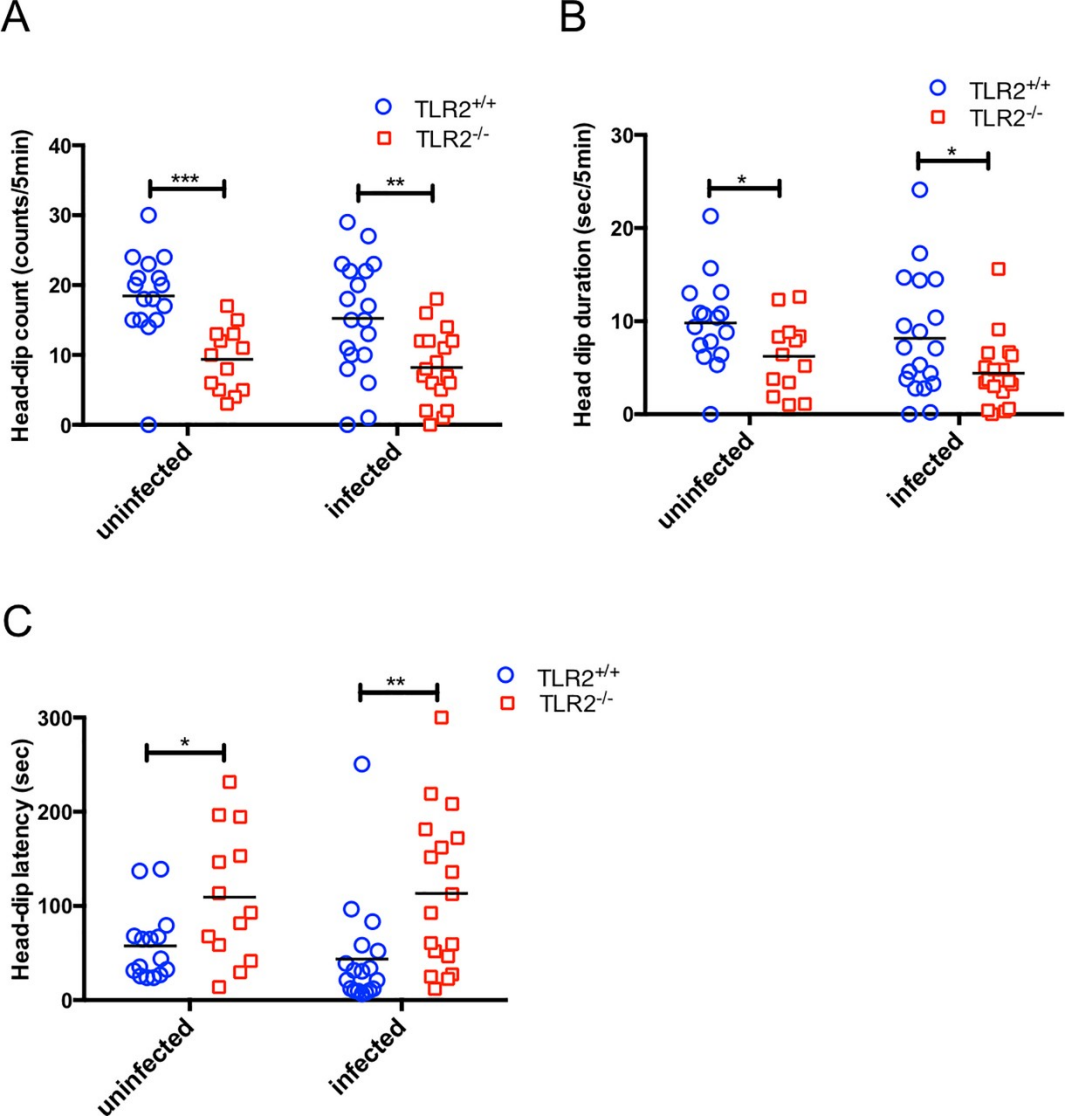
Effects of *T*. *gondii* infection on anxiety-related behavior in mice. Changes in the exploratory behavior at 31 days post infection: (A) head-dip count (counts/5 min), (B) head-dip duration (sec/5 min), and (C) head dip latency (sec). Data were summarized from two independent experiments (uninfected TLR2^+/+^, n = 16; uninfected TLR2^-/-^, n = 13; infected TLR2^+/+^, n = 21; infected TLR2^-/-^, n = 18). Significant differences among the four groups were analyzed by a two-way ANOVA followed by t tests (*, *p* < 0.05; ***, *p* < 0.01; ****, *p* < 0.001), significant main effects are shown for (A) TLR2 deficiency [*F*_(1,62)_ = 25.30, *p* < 0.0001], (B) TLR2 deficiency [*F*_(1,62)_ = 8.996, *p* < 0.01], and (C) TLR2 deficiency [*F*_(1,60)_ = 13.83, *p* < 0.001]. The interaction between *T*. *gondii* infection and TLR2 deficiency was not statistically significant.

The hole-board test is generally used to assess anxiety in rodents. Head-dipping behavior in this test reflects the anxiolytic state of the animals [28]. The head-dipping number, and head-dip duration of the uninfected TLR2^-/-^ mice decreased and head-dipping latency of the uninfected TLR2^-/-^ mice increased compared with the uninfected TLR2^+/+^ mice (Fig 2). Similar results were obtained in the infected TLR2^+/+^ and TLR2^-/-^ mice (Fig 2). The two-way ANOVA analysis showed that the factor ‘TLR2 deficiency’ was the statistically significant factor affecting mouse behavior in the hole-board test, while no significant effects for the factor ‘*Toxoplasma* infection’ and no interaction between ‘*Toxoplasma* infection’ and ‘TLR2 deficiency’ were observed. These results indicate that increased anxiety levels were present in the TLR2^-/-^ mice.

### Fear-conditioning testing on *T*. *gondii*-infected and uninfected mice

**Fig 3:**
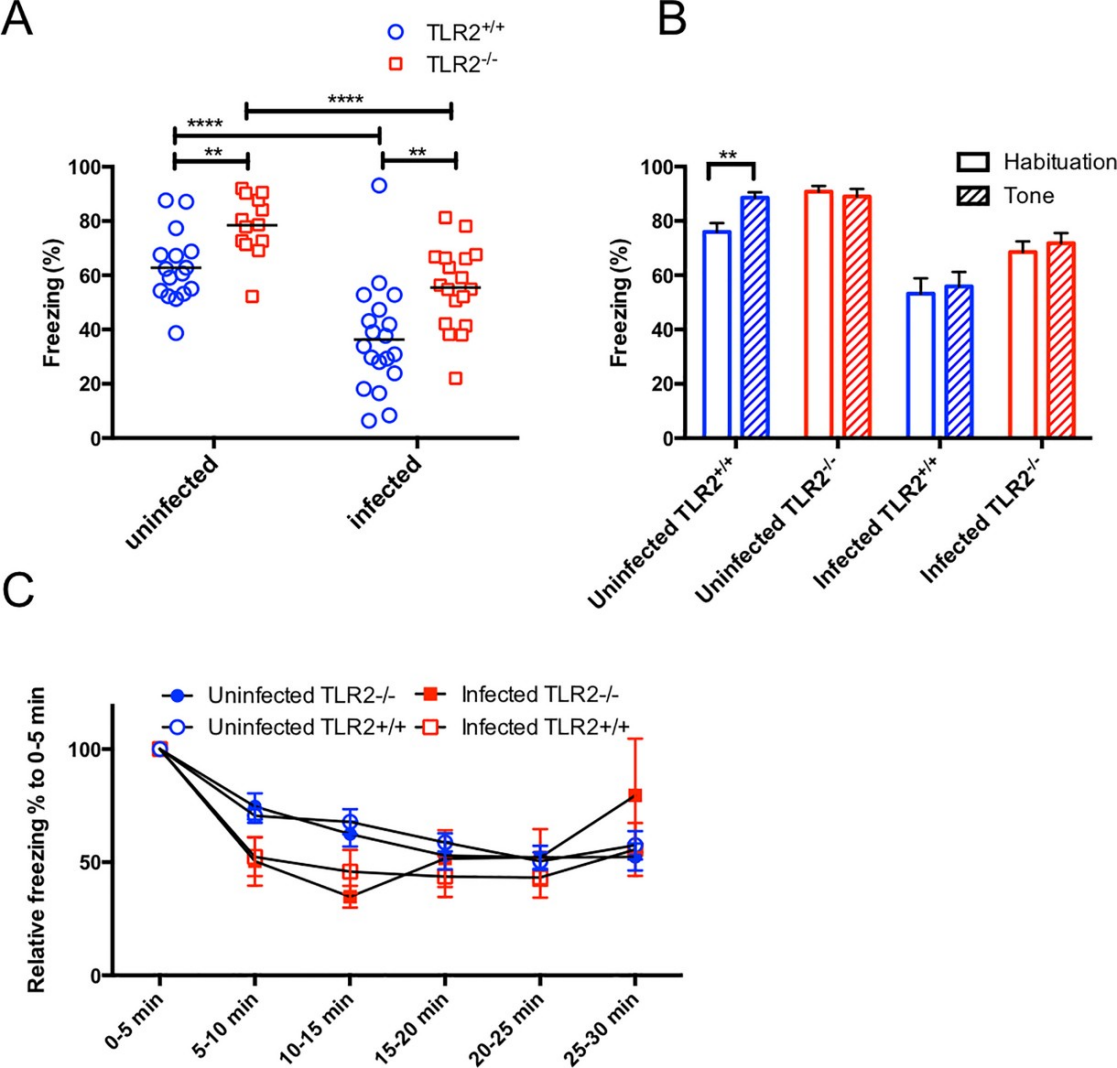
Effects of *T*. *gondii* infection on fear memory consolidation in mice. The ordinate shows the percentage of time spent freezing. (A) Contextual conditioned freezing time. Significant differences among the four groups were analyzed by a two-way ANOVA followed by t tests (**, *p* < 0.01; ***, *p* < 0.001; ****, *p* < 0.0001), significant main effects are shown for TLR2 deficiency [*F*_(1,62)_ = 20.06, *p* < 0.0001] and *T*. *gondii* infection [*F*_(1,62)_ = 40.58, *p* < 0.0001]. The interaction between *T*. *gondii* infection and TLR2 deficiency was not statistically significant. (B) Tone-conditioned freezing time. Significant differences between habituation and tone sessions were determined by unpaired *t* tests (**p < 0.01). (C) Time course for fear extinction as revealed by the decreased freezing response in the extinction test. Statistical analysis between experimental group pairs including uninfected TLR2^+/+^ mice and uninfected TLR2^-/-^ mice, uninfected TLR2^+/+^ mice and infected TLR2^+/+^ mice, and uninfected TLR2^-/-^ mice and infected TLR2^-/-^ mice were performed by a two-way ANOVA but no statistically significant main effects or interactions were observed. The average freezing response percentage across the 5 min intervals in the extinction test was normalized to the first 5 min. Freezing was calculated by dividing the freezing time by the observation times (300 s) in the context test, habituation (180 s), and tone (180 s) during the tone test, and over 5 min for every 5 min interval in the extinction test. Data represent the means ± SEMs. Data are summarized from two independent experiments (uninfected TLR2^+/+^, n = 16; uninfected TLR2^-/-^, n = 13; infected TLR2^+/+^, n = 21; infected TLR2^-/-^, n = 18).

We have previously reported that there is impaired fear memory consolidation in mice infected with *T*. *gondii* [22]. In the present study, we examined whether TLR2 deficiency was associated with this memory deficit. However, unexpectedly, we found that mouse freezing behavior in the context test increased significantly, but this was induced by TLR2 deficiency in its own right (Fig 3A). In fact, infection with *T*. *gondii* decreased the time spend in freezing activity in both TLR2^+/+^ and TLR2^-/-^ mice in the context test (Fig 3A). Our two-way ANOVA analysis of the context test showed significant effects for the factors ‘*Toxoplasma* infection” (*p* < 0.0001) and ‘TLR2 deficiency’ (*p* < 0.0001), but there was no interaction between the two factors (*p* = 0.6465).

In the tone test, when a mouse normally memorizes an association between conditioned tone and foot shocks, it shows an increased freezing response when it is re-exposed to the conditioned tone. Although the freezing reactions of the uninfected TLR2^+/+^ mice increased after re-exposing them to the conditioned tone, the other groups did not show such a change in reaction (Fig 3B). These results suggest that the tone conditioned fear memory response was impaired by both TLR2 deficiency and *T*. *gondii* infection, but no significant interaction was observed between these factors.

Finally, the effects of ‘*T*. *gondii* infection’ and ‘TLR2 deficiency’ on the extinction of contextual fear memory were evaluated in the extinction test (Fig 3C). In the first 0–5 min of the test, the percentage of infected mice experiencing freezing behavior decreased significantly compared with the percentage for the uninfected mice (data not shown). This difference might reflect the conditioned context results. Therefore, to evaluate the extinction of fear memory, the average percent of the freezing response across every 5 min of the extinction test was normalized to that of the first 5 min. The data represent the transition of the extinction phase of the conditioned fear. The two-way ANOVA analyses showed no significant main effects and interaction (Fig 3C). These results suggest that both TLR2 deficiency and *T*. *gondii* infection did not affect the extinction of the conditioned fear memory.

### Histopathological analysis and parasite numbers in the mouse brains

**Fig 4:**
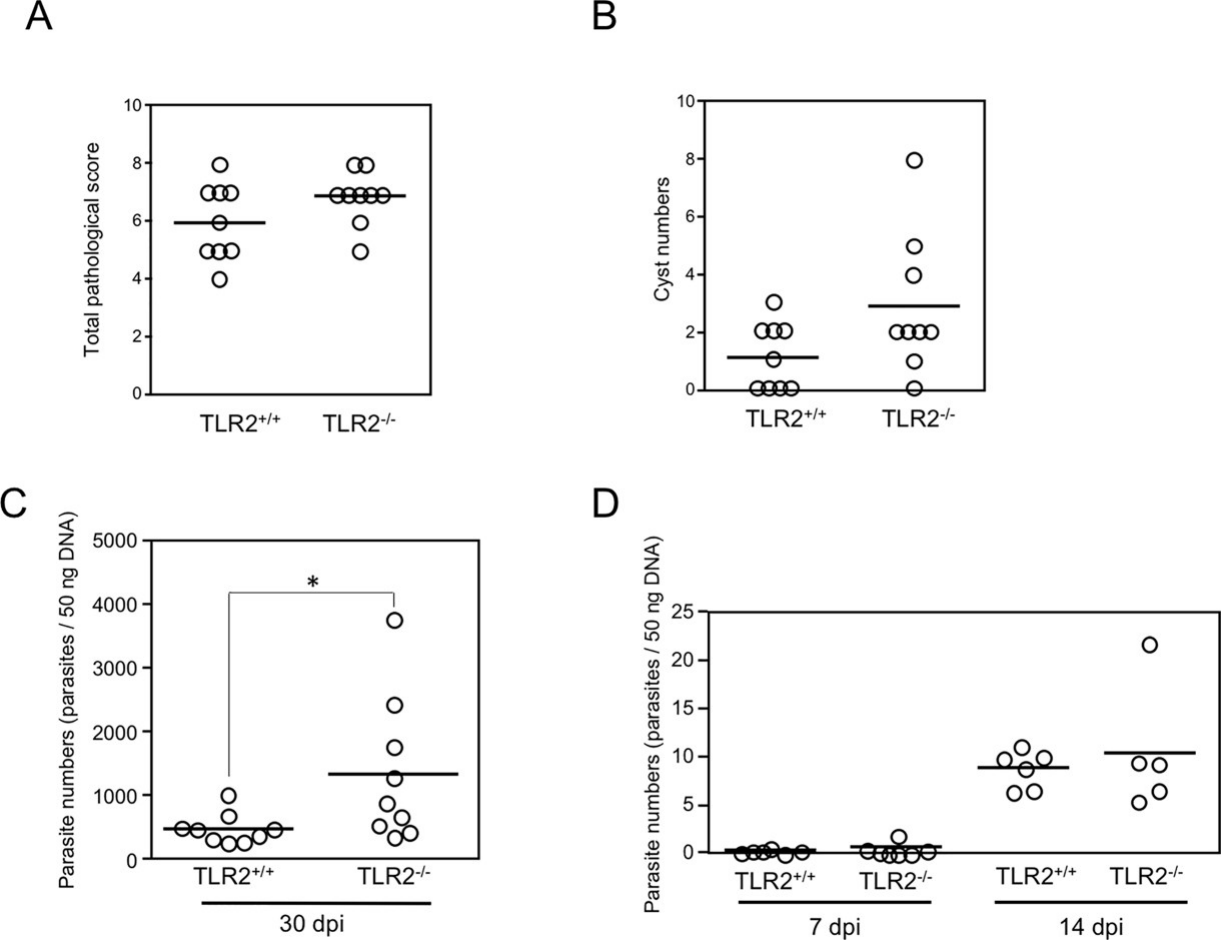
Histopathological lesions and parasite burdens in the brains of *T*. *gondii*-infected mice. On day 30 after infection, brain samples were collected. The total pathological score (A) and number of brain cysts (B) were calculated. Each circle represents the data for one mouse, and the bars represent the average values for all the data points in two independent experiments (TLR2^+/+^ mice, n = 9; TLR2^–/–^mice, n = 9). There was no statistically significant difference between the two groups in the unpaired *t* test (*p = 0.0628). (C) On day 30 after infection, brain samples were collected. DNA was extracted from each brain sample and the parasite numbers were quantified. Each circle represents the data for one mouse, and the bars represent the median value from all the data points in two independent experiments (TLR2^+/+^ mice, n = 9; TLR2^–/–^mice, n = 9). Statistically significant differences were determined by the Mann-Whitney test (*p = 0.0315). (D) TLR2^+/+^ mice and TLR2^–/–^mice were infected with *T*. *gondii* tachyzoites. On day 7 and 14 after infection, brain samples from all the mice were collected. DNA was extracted from each sample and the parasite numbers were quantified. Each circle represents the data for one mouse, and the bars represent the average values for all the data points in one experiment (TLR2^+/+^ mice, n = 6; TLR2^–/–^mice, n = 5). Significant differences were determined by unpaired *t* tests (*p < 0.05).

Next, we investigated the role played by TLR2 in *T*. *gondii* infections in terms of parasite pathology. Histopathological analysis showed that the *T*. *gondii* infections induced brain lesions in the mice, such as inflammatory cell infiltration, but the severity of the brain lesions did not differ significantly between the infected TLR2^+/+^ and infected TLR2^-/-^ mice (*p* = 0.469) (Fig 4A). However, cyst numbers in the brains of the infected TLR2^-/-^ mice were slightly higher than those of the infected TLR2^+/+^ mice, but there was no significant difference (*p* = 0.0628) (Fig 4B). Our quantitative real-time PCR analysis showed that the number of parasites in the brains of the TLR2^-/-^ mice was significantly higher than that of the TLR2^+/+^ mice at 30 days after infection (*p* = 0.0315, Fig 4C). In contrast, the numbers of brain-located parasites did not differ between the TLR2^+/+^ and TLR2^-/-^ mice at 7 and 14 days after infection (*p* = 0.3315 and *p* = 0.5787, respectively) (Fig 4D).

### iNOS and cytokine mRNA expression in the brains of *T*. *gondii*-infected TLR2^+/+^ and TLR2^–/–^mic

**Fig 5:**
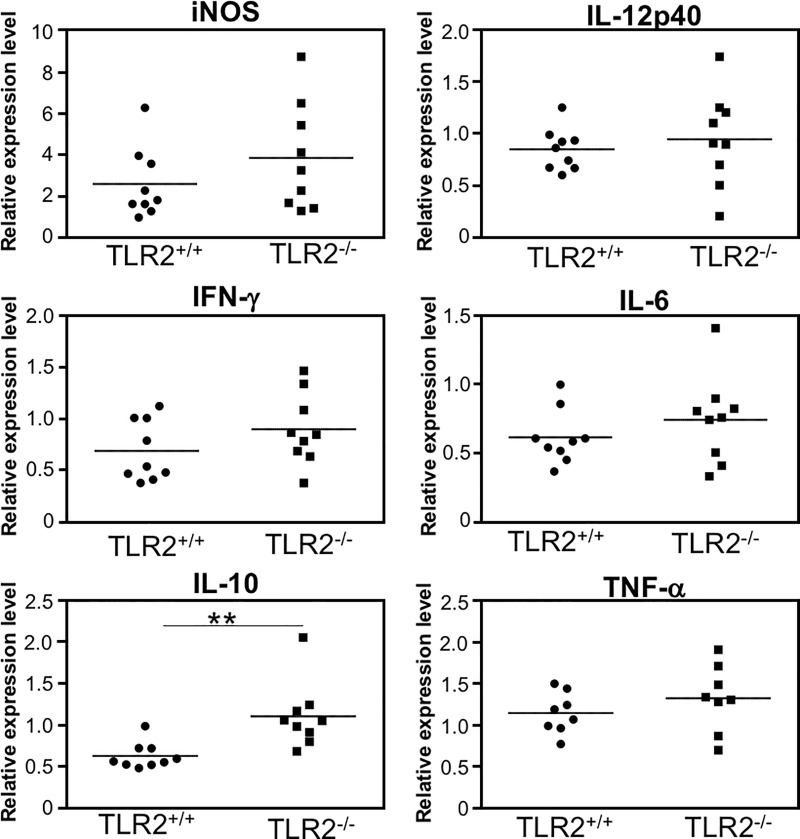
iNOS and cytokine mRNA expression in the brains of *T*. *gondii*-infected TLR2^+/+^ and TLR2^–/–^mice. TLR2^+/+^ and TLR2^–/–^mice were infected with *T*. *gondii* tachyzoites. On day 30 after infection, brain samples from all the mice were collected. Total RNA was extracted from each sample and iNOS and cytokine mRNA expression was quantified by real-time PCR. Each circle and square represent the data for one mouse, and the bars represent the average values of all the data points in two independent experiments (TLR2^+/+^ mice, n = 9; TLR2^–/–^mice, n = 9). Significant differences were determined by unpaired *t* tests (*p < 0.05).

In general, TLR2 activates the nuclear factor-kB (NF-kB) signal transduction cascade, which leads to the production of a variety of inflammatory mediators and cytokines. To clarify the role of TLR2 in the brain, we examined iNOS and cytokine mRNA expression in the brain tissue (Fig 5). IL-10 expression levels were higher in the brains from the infected TLR2^-/-^ mice than those of the infected TLR2^+/+^ mice (*p* < 0.01). In contrast, no differences were observed in the expression levels of iNOS and other cytokines in the infected TLR2^+/+^ and infected TLR2^-/-^ mice at 30 dpi (iNOS, *p* = 0.2412; IFN-γ, *p* = 0.1846; IL-12p40, *p* = 0.5666; IL-6, *p* = 0.3212; TNF-α, *p* = 0.299).

## Discussion

We initially investigated whether TLR2 deficiency itself would affect behavior. Uninfected TLR2^-/-^ mice showed an increase in latency to the first movement in the open-field test. Moreover, TLR2 deficiency decreased both the number and duration of head-dips and increased the latency to head-dipping period in the hole-board test. These observations in the uninfected TLR2^-/-^ mice are explainable by more cautious locomotion in view of their higher anxiety levels in the novel situation [29].

In the fear-conditioning test with the uninfected mice, in contrast to our initial expectation, our results revealed that the TLR2^-/-^ mice experienced increased freezing behavior during the time spent in the conditioned context. Moreover, in the tone test, freezing during the conditioned tone presentation did not increase in the uninfected TLR2^-/-^ mice. Contrasting with the present study, a previous report showed that TLR2^-/-^ mice showed decreased freezing activity during cued and contextual conditioning [3]. This behavioral difference possibly stems from the different experimental conditions between these studies (e.g. the electrical shock strength, and the number of foot shocks applied). A previous study also showed that TLR2 deficiency in mice was able to impair hippocampal neurogenesis [2]. Other studies have reported that the reduction in neurogenesis in the hippocampus coincides with impaired learning and memory [30–33]. Moreover, TLR2^-/-^ mice experience increased cell death in the various brain regions such as the hippocampus [3]. Thus, these observations support an inhibitory role for TLR2 deficiency in hippocampus-dependent memory. Therefore, in our study, the higher anxiety levels observed in the TLR2^-/-^ mice in the hole-board test might have heightened their freezing behavior in the contextual conditioned situation rather than enhancing the hippocampus-dependent memory processes *per se*. However, how TLR2 interacts with hippocampal memory processes remains elusive in terms of its mechanism.

Next, we discuss the behavioral changes caused by *T*. *gondii* infection during the subacute stage. There was no consistent indication that infection with *T*. *gondii* affected the anxiolytic state and the locomotor activity of the mice. This observation may reflect the use of different *T*. *gondii* strains, different host species and sexes and/or different methodologies to measure behavior in the mice [16]. There is a discrepancy regarding the effect of *T*. *gondii* infection on motor activity. It has been found that general motor activity increases, diminishes, or is unaffected in *T*. *gondii* infections [17,34–38]. The observation of decreased locomotor activity in the open-field test is suggestive of increased anxiety levels in the animals [39]. However, in the present study, both *T*. *gondii*-infected TLR2^+/+^ and TLR2^-/-^ mice experienced decreased bodyweights, and average speeds. Therefore, we assume that the decreased locomotor activity we observed may reflect the general state of health of the mice.

The effect of latent *T*. *gondii* infection in the hole-board test, as reported previously, showed unchanged exploration of male mice infected with *T*. *gondii* [40], and our results are consistent with this result. In the fear-conditioning test, *T*. *gondii* infection impaired contextual and tone-associated fear memory. The initial aim of our study was to examine whether the absence of TLR2 signaling affected the behavioral changes caused by *T*. *gondii* infection or not. However, throughout the behavioral tests, there was no interaction between ‘*T*. *gondii* infection’ and ‘TLR2 deficiency’. These results suggest that infection with *T*. *gondii* impaired the ability to consolidate the fear memory in the mice, but this occurred in a TLR2-independent manner, at least under our experimental conditions.

In the present study, the TLR2^-/-^ mice experienced an increased parasite burden at 30 days, but not at 7 and 14 days after intraperitoneal infection with 1 × 10^3^ PLK strain tachyzoites. Although we cannot exclude the existence of DNA from dead parasites in the real-time PCR results, we found an increased (but not significant) number of cysts in the brains from the TLR2^-/-^ mice, suggesting an increased burden with viable parasites. Although the mechanism concerning how *T*. *gondii* reaches the brain has not been elucidated in detail, *T*. *gondii* crosses the blood-brain barrier during the acute stage of its infection [41–43]. In this study, parasite DNA was detected in the brain tissues from both TLR2^+/+^ and TLR2^-/-^ mice during the acute stage (14 dpi), but there was no significant difference in the parasite counts between them. This finding suggests that TLR2 deficiency did not cause early dysfunction of the blood-brain barrier. A previous study has also reported that TLR2 deficiency did not affect the parasite distribution in the peripheral organs of the experimental mice, at least when a non-lethal parasite dose was used [5].

Our previous study showed that production of IL-6 and prostaglandin E2 in astrocytes and IL-12p40, IL-6, IL-10 and IL-1β in microglia were much or completely impaired when the TLR2 gene was absent, suggesting a pivotal role for TLR2 in the protective host response in the CNS [9]. Thus, the impaired activities of brain cells may have resulted in increased parasite numbers in the brains of the TLR2^-/-^ mice at 30 days post infection. However, in the present study, any differences in the severity of the histopathological lesions and the expression levels of inflammatory cytokines, except for IL-10, were not observed between the infected TLR2^+/+^ and infected TLR2^-/-^ mice. Moreover, although differences in the immunological status of the uninfected TLR2^+/+^ and the uninfected TLR2^-/-^ mice were not investigated in this study, previous studies have reported that TLR2^-/-^ mice have normal thymocyte and splenocyte compositions, and their surface expression of B220, IgM, and IgD on splenocytes are almost identical to those of the wild-type mice [23]. Moreover, their serum cytokine levels (e.g., IFN-γ, TNF-α, IL-4, and IL-2) were not altered in TLR2^-/-^ mice compared with the wild-type C57BL/6 mice [44]. These results suggest that TLR2 deficiency shows no obvious adverse in its effects on the basal immunological profile of the mice. Thus, contrary to our initial expectations, our data indicate that TLR2 does not contribute strongly to the immune-pathological changes occurring during the subacute stage of infection with *T*. *gondii*, under our experimental conditions at least.

## Conclusions

To the best of our knowledge, this is the first study to investigate the effects of TLR2 deficiency on latent *Toxoplasma* infections in mice. The TLR2^-/-^ mice we tested showed increased baseline anxiety-like behavior. In addition, our results suggest there was an impairment of fear memory caused by *T*. *gondii* infection in a TLR2-independent manner, at least under our experimental conditions. In contrast to our initial expectation, this study has revealed that TLR2 does not play a dominant role in the brain protective immune response to latent *T*. *gondii* infection, with the exception of the parasite burden in the brain.

However, it should be pointed out that conventional (whole-body) knockout^-^ mice were used in this study. Whole-body TLR2 deficiency, which causes complicated changes in mouse behavior, makes it difficult to understand the exact role played by TLR2-signaling during chronic *T*. *gondii* infection of the brain. Further studies using a better model, for example mice with a conditional deletion of TLR2 in their microglial cells and astrocytes or identification of the TLR2 ligand in *T*. *gondii* should extend our understanding of the role of TLR2 in cerebral toxoplasmosis.

## Supporting information

S1 Fig Histopathological lesions and cysts in *T*. *gondii*-infected mouse brains detected by hematoxylin–eosin (HE) staining. Representative example of histopathological lesion in the brain tissue from a *T*. *gondii*-infected mouse, score 1: localized mild perivascular cuffs, score 2: moderate glial cell infiltration, score 3: severe inflammatory cell infiltration, and a *T*. *gondii* tissue cyst in the brain. TLR2^+/+^ mice and TLR2^–/–^mice were infected with *T*. *gondii* tachyzoites. On day 30 after infection, brain samples were collected.
(PDF)

S2 Fig Analysis of bodyweight changes. Relative body weight changes in the mice were recorded until 28 days post infection. Data are the mean values ± SD for all the mice in each group that were used in two independent experiments (uninfected TLR2^+/+^, n = 16; uninfected TLR2^-/-^, n = 13; infected TLR2^+/+^, n = 21; infected TLR2^-/-^, n = 18). Significant differences were determined by a two-way ANOVA and post hoc Tukey’s test, and both the infected TLR2^+/+^ and infected TLR2^-/-^ mice showed significant body weight losses from day 9 post infection (*p < 0.05).
(PDF)

S1 Table Minimal data set. (XLSX)
