# Supplementary material for: Involvement of Toll-like receptor 2 in the cerebral immune response and behavioral changes caused by latent Toxoplasma infection in mice
Source: PLoS One. 2019 Aug 12;14(8):e0220560. doi: 10.1371/journal.pone.0220560 (PMC6690529; doi:10.1371/journal.pone.0220560)
Supplement: S1 Fig — Representative example of histopathological lesion in the brain tissue from a T. gondii-infected mouse, score 1: localized mild perivascular cuffs, score 2: moderate glial cell infiltration, score 3: severe inflammatory cell infiltration, and a T. gondii tissue cyst in the brain. TLR2+/+ mice and TLR2–/–mice were infected with T. gondii tachyzoites. On day 30 after infection, brain samples were collected. (PDF) [file pone.0220560.s001.pdf]

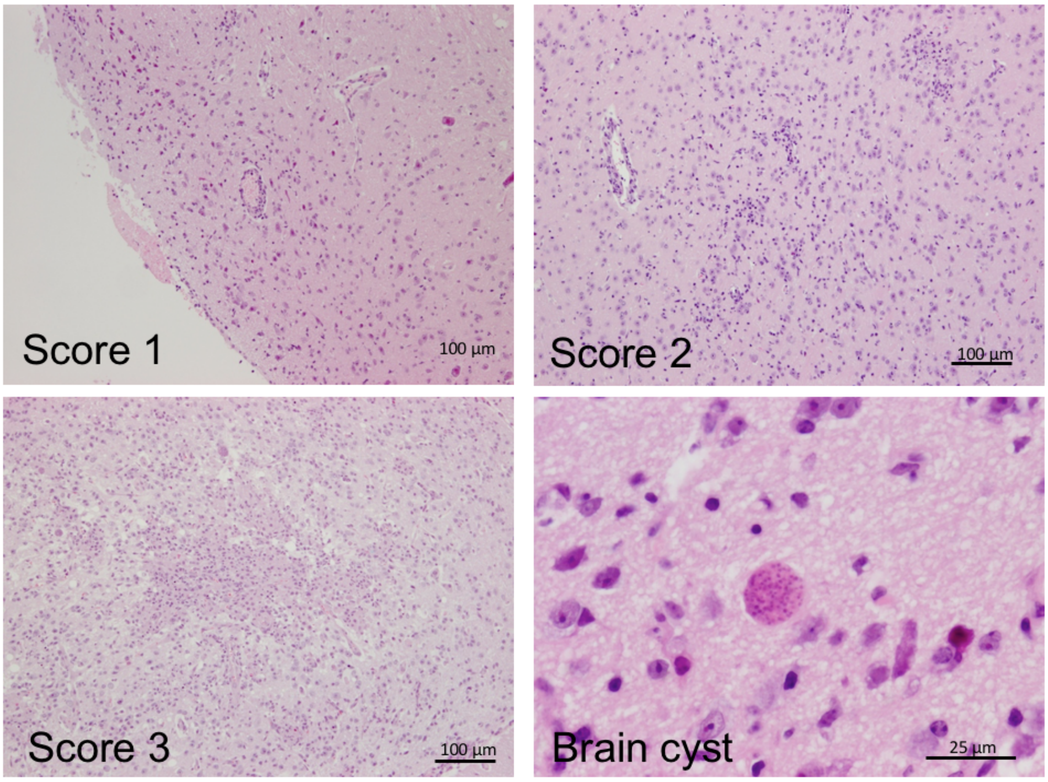

S1 Fig. Histopathological lesions and cysts in *T. gondii*-infected mouse brains detected by hematoxylin–eosin (HE) staining. Representative example of histopathological lesion in the brain tissue from a *T. gondii*-infected mouse, score 1: localized mild perivascular cuffs, score 2: moderate glial cell infiltration, score 3: severe inflammatory cell infiltration, and a *T. gondii* tissue cyst in the brain. TLR2$^{+/+}$ mice and TLR2$^{-/-}$ mice were infected with *T. gondii* tachyzoites. On day 30 after infection, brain samples were collected.
