# Supplementary material for: Involvement of Toll-like receptor 2 in the cerebral immune response and behavioral changes caused by latent Toxoplasma infection in mice
Source: PLoS One. 2019 Aug 12;14(8):e0220560. doi: 10.1371/journal.pone.0220560 (PMC6690529; doi:10.1371/journal.pone.0220560)
Supplement: S2 Fig — Relative body weight changes in the mice were recorded until 28 days post infection. Data are the mean values ± SD for all the mice in each group that were used in two independent experiments (uninfected TLR2+/+, n = 16; uninfected TLR2-/-, n = 13; infected TLR2+/+, n = 21; infected TLR2-/-, n = 18). Significant differences were determined by a two-way ANOVA and post hoc Tukey’s test, and both the infected TLR2+/+ and infected TLR2-/- mice showed significant body weight losses from day 9 post infection (*p < 0.05). (PDF) [file pone.0220560.s002.pdf]

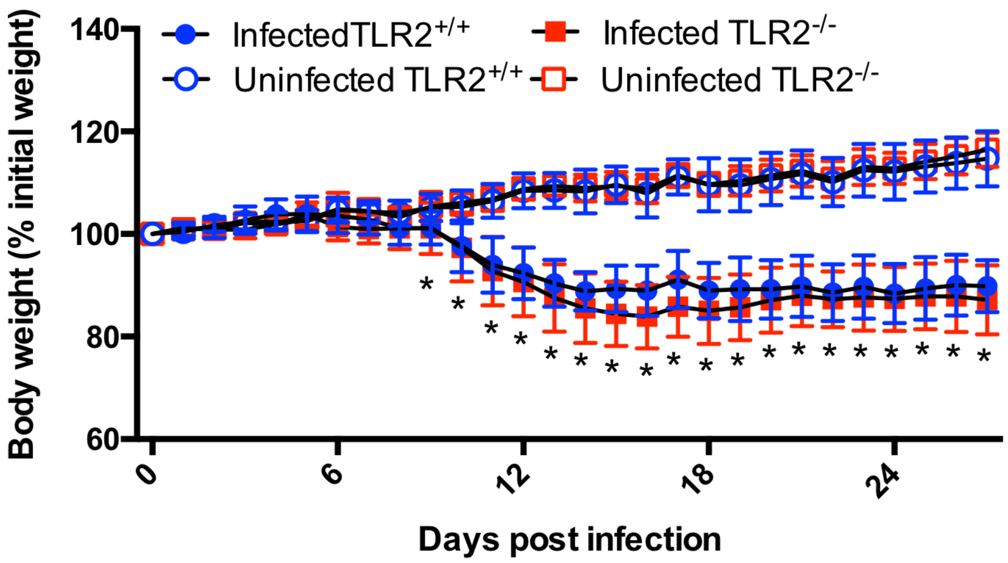

S2 Fig. Analysis of bodyweight changes. Relative body weight changes in the mice were recorded until 28 days post infection. Data are the mean values ± SD for all the mice in each group that were used in two independent experiments (uninfected TLR2$^{+/+}$, n = 16; uninfected TLR2$^{-/-}$, n = 13; infected TLR2$^{+/+}$, n = 21; infected TLR2$^{-/-}$, n = 18). Significant differences were determined by a two-way ANOVA and post hoc Tukey's test, and both the infected TLR2$^{+/+}$ and infected TLR2$^{-/-}$ mice showed significant body weight losses from day 9 post infection (*$p < 0.05$).
